# A novel fractional micro-plasma radio-frequency technology for the treatment of facial scars and rhytids: A pilot study

**DOI:** 10.3109/14764172.2010.514921

**Published:** 2010-09-09

**Authors:** Shlomit Halachmi, Arie Orenstein, Tania Meneghel, Moshe Lapidoth

**Affiliations:** 1 Laser Unit, Department of Dermatology, Rabin Medical Center, Petach Tikva, Israel; 2 Department of Plastic Surgery, Chaim Sheba Medical Center, Tel Hashomer, Israel; 3 Renaissance Medical Center for Prevention and Rejuvenation, Jardin Girassol Americans, Sao Paulo, Brazil; 4 Sackler School of Medicine, Tel Aviv University, Tel Aviv, Israel

**Keywords:** Acne, fractional, plasma, radio frequency, scars, skin resurfacing

## Abstract

*Introduction:* Fractional ablative and non-ablative lasers have gained popularity in the treatment of acne scars and rhytids due to their efficacy and improved tolerability. Plasma and radio frequency (RF) have also emerged as methods for ablative or non-ablative energy delivery. We report preliminary experience with a novel fractional micro-plasma RF device for the treatment of facial acne scars and rhytids. *Methods:* Sixteen patients with facial acne scars or rhytids were treated at 4-week intervals. Treatment parameters were titrated to an immediate end point of moderate erythema. The clinical end point for cessation of treatment was the attainment of satisfactory clinical results. Results were monitored photographically up to 3 months after treatment. *Results:* Acne scars showed marked improvement after two to four treatments. Facial rhytids demonstrated reduced depth after two treatments and marked improvement after four treatments. Treatment was well tolerated by all participants, with transient erythema and short downtime. These results provide initial evidence for the safety and effectiveness of fractional micro-plasma RF as a low-downtime and well-tolerated modality for the treatment of acne scars and facial rhytids.

## Introduction

Fractional skin resurfacing, primarily with erbium and carbon dioxide (CO_2_) lasers, has emerged as an effective therapeutic approach with an attractive efficacy-to-downtime ratio (1-4). By limiting energy to a grid of small foci within a treatment field, high energies can be delivered, while undamaged skin surrounding each focus of ablation provides the basis for rapid re-epithelialization. Fractional lasers have been applied to the treatment of acne scars and facial rhytids with great success (5,6). Plasma technology has also been reported to be safe and effective when applied in a non-fractional manner for facial rejuvenation, albeit with prolonged erythema and a need for pain control when used at higher energies (7-10). More recently, devices have been developed that allow the treatment of the face with fractional radio-frequency (RF), based also on the rationale of placing a grid of high-energy foci evenly on the skin.

**Figure 1:**
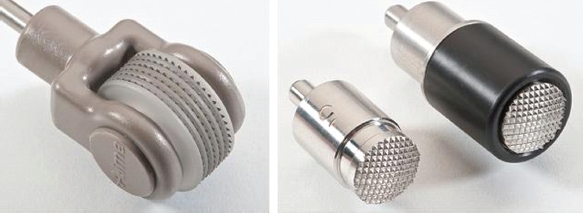
Pixel RF tips, showing the spicules that form the small foci of contact with the skin, in the roller confi guration (left) and in stationary tips (right).

**Figure 2:**
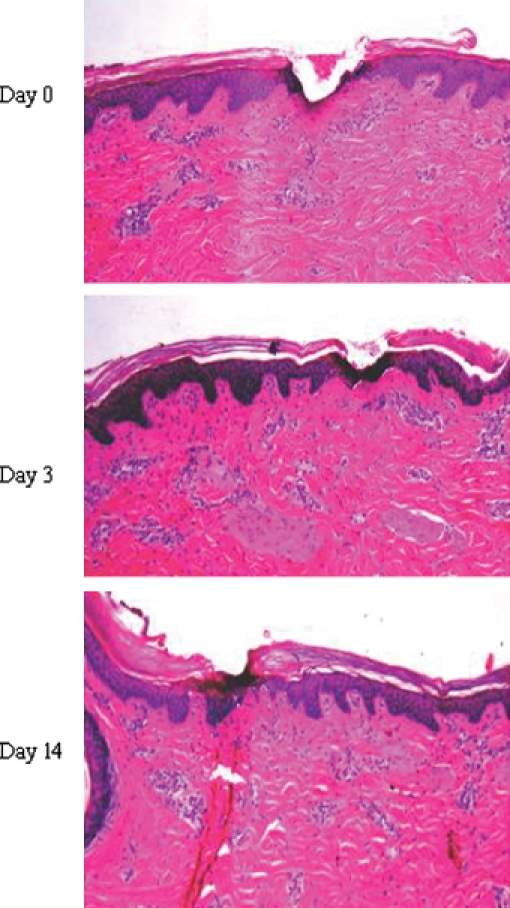
Histological changes following Pixel RF application to porcine skin in preclinical studies. Day 0: wedge-shaped channel of tissue ablation formed in the epidermis and papillary dermis by a single pulse of 45 watts RF; Day 3: re-epithelialization of full-thickness epidermis, except the stratum corneum; Day 14: complete healing.

The Pixel RF device (Alma Lasers, Israel) was developed as a minimally ablative fractional technology, which uses unipolar RF technology to provoke plasma sparks, creating multiple controlled micro-perforations on the skin. The handpieces give rise to a series of closely spaced spicules, which contact the skin and provide a thin air gap between the skin surface and the roof of the electrode (Figure 1). The discharge of RF energy at a small distance from the skin forms plasma, a gas-like state in which a portion of the molecules are ionized. As plasma is very sensitive to electromagnetic fields, the RF current triggers micro-sparks in the plasma between the skin surface and the electrode spicules. These sparks cause mild epidermal ablation and perforate the dermis superficially to form micro-channels. Porcine skin models, performed prior to clinical evaluation, demonstrated that the depth and diameter of the ‘pixels’ of injury depend on the RF power and pulse duration, and range from 100 to 150 mm in depth and from 80 to 120 mm in diameter (Figure 2). The technology improves upon conventional RF by leveraging the generation of plasma to generate focally higher con-ductivity and thereby triggering micro-sparks and high temperatures in minute foci.

We report here the initial experience of treating facial acne scars and rhytids.

## Materials and methods

All participants signed an informed consent prior to treatment. Indications included facial acne scars and facial rhytids. Contraindications to treatment were Fitzpatrick skin types V-VI, active bacterial or viral infection, use of isotretinoin within the prior 12 months, impaired immune function, active collagen vascular disease, malignancy, diabetes, pregnancy, a pacemaker or defibrillator, metal implants near the treatment area, and an ablative skin procedure within the prior 3 months. At one clinical site (T.M.), patients who were known to be susceptible to HSV reactivation received prophylaxis with oral antiherpetic medications on the day before treatment (acyclovir 400 mg twice daily or famciclovir 250 mg twice daily). At the same clinical site, patients felt to be at increased risk for hyperpigmentation, based on physician assessment, were pre-treated with hydroquinone starting 1 month prior to treatment.

After pre-treatment with a topical lidocaine gel, the Pixel RF device was applied using a 12 mm stationary tip or a roller tip (Figure 1). For acne scars, Fitzpatrick skin types I—III were treated with the 12-mm stationary tip for five stacked pulses at 40-50 watts, 0.2 seconds followed by five passes of the roller tip at 35-50 watts over the entire anatomical facial unit. Fitzpatrick IV skin was treated with five to seven passes of the roller tip only, at 35-50 watts. For facial rhytids, all skin types were treated with the 12-mm stationary tip for five stacked pulses at 40-50 watts, 0.2 seconds over the rhytids, followed by five to six passes of the roller tip at 35-50 watts over the affected facial units.

Treatments were repeated at intervals of 4-6 week. The clinical end point for cessation of treatment was the attainment of satisfactory clinical results.

## Results

A total of 16 patients were treated (five male, 11 female). The clinical endpoint for completion of treatment was moderate erythema. Owing to the rapid motion of the roller tip over large areas, the five passes over the face were swift and well tolerated. The total treatment time for the full-face was approximately 10 minutes. All facial treatments were well tolerated with no adverse events. Mild-to-moderate erythema was present for 1 day after treatment. A pale pixel-pattern grid was evident for 3 days, over a background of fine exfoliative scale.

**Figure 3:**
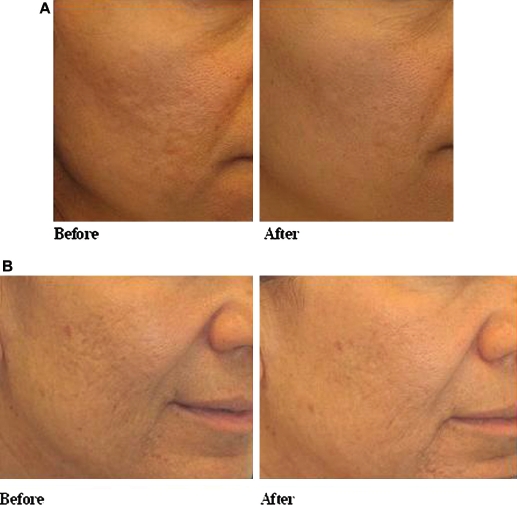
Treatment of acne scars. (A) Acne scars before and 1.5 months after three treatments; (B) acne scars before and 3 months after three treatments.

Eight patients with acne scars were treated. The mean age was 34.5 years (range 23-57). Patients underwent one to four treatments (mean 3.1). Acneiform scars demonstrated cumulative improvement, with notable improvement after three treatments (Figure 3A). The effect was maintained at 3 months’ follow-up (Figure 3B). Of interest, patients with mild active acne experience a reduction in active acneiform lesions during the treatment and follow-up interval.

**Figure 4:**
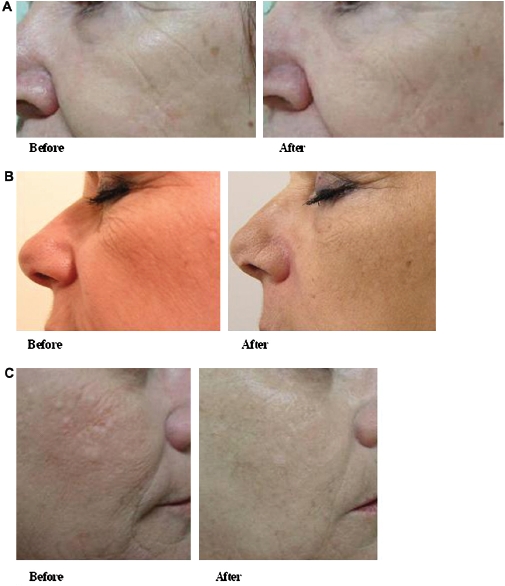
Treatment of facial rhytids. (A) Facial rhytids before and 1 month after two treatments; (B) facial rhytids before and 1 month after three treatments; (C) facial rhytids before and 1 month after four treatments.

**Figure 5:**
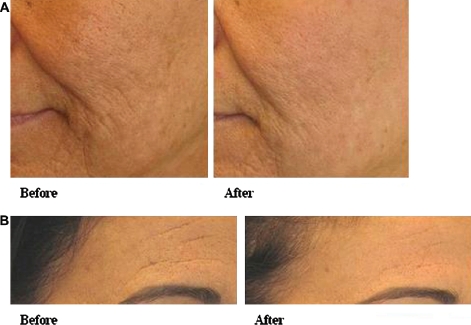
Treatment of facial rhytids. (A, B) Facial rhytids before and 3 months after three treatments.

Eight patients underwent treatment for facial rhytids. The mean age was 58.8 years (range 51-63). Patients underwent two to four treatments (mean 2.6). Wrinkles were visibly reduced after two treatments, with notable improvement in wrinkle appearance and visual skin texture after four treatments (Figure 4). Longer-term follow-up showed maintained benefit at 3 months, with a more even appearance as well as reduction in the visual prominence of wrinkles (Figure 5).

## Discussion

In this preliminary assessment, the Pixel RF demonstrated visible results in the improvement of facial acne scars and rhytids after a series of treatments. Incremental benefit was achieved with repeated treatments, and results were maintained at 3-month follow-up evaluations. No severe adverse events were reported.

Of interest, in several participants, incidental benefits were noted outside of the indication being treated. In one participant, treatment of acne scars resulted in improvement of active acneiform lesions within the treatment area, presumably due to dermal heating and its effects on sebaceous gland function, although the mild exfoliative response to treatment within the first 3 days after the procedure may contribute as well. In another participant, treatment of acne scars showed improvement in freckling within the treated area. Such a response is compatible with epidermal turnover and subsequent rejuvenation as a response to the fractional micro-plasma RF, and suggests that the procedure may be a good treatment option for reversing certain epidermal signs of photoaging, in addition to targeting dermal deficits. This would make the procedure appropriate not only for discrete lesions, as was assessed here, but for improving the appearance of entire regions. Given the rapid coverage with the roller tip, which provides treatment of the full face with five passes in 10 minutes, the procedure may be amenable to the treatment of larger areas on the body. This will be investigated in future studies.

Taken together, the results provide evidence for safety and for beneficial effects of fractional micro-plasma RF treatments in visible improvement of facial acne scars and rhytids. The Pixel RF adds an additional tool to the growing armamentarium of fractional therapies.
